# An Unsuccessful Case of Acute Spinal Meningitis

**Published:** 1858-04

**Authors:** Ross C. Russ

**Affiliations:** Royalton, Indiana


ARTICLE V.

### AN UNSUCCESSFUL CASE OF ACUTE SPINAL MENINGITIS.

BY DR. ROSS C. RUSS, OF ROYALTON, INDIANA.

Why not sometimes report the unsuccessful as well as the successful cases in our practice?

I was summoned, on the morning of the 4th of January, 1858, to Jasper Montgomery, aged three years.

PRESENT CONDITION.—I found him lying upon his left side, with rigid contraction of the muscles along the spinal column; the head drawn backwards towards the sacrum, in the form of an arch—in technical language, opisthotonos; tenderness, on pressure, over the region of the cervical vertebræ; excruciating pain, when attempted to move in bed; tongue coated with a yellowish white fur, with redness around the tip and edges; hurried respiration; pulse about 100 beats to the minute; hot skin; anorexia; bowels somewhat constipated, etc.

Previous History.—The mother stated her child had previously enjoyed very good health, until about a week since. He complained of pain in his back, walked with some degree of difficulty, and was seized with retching and vomiting, when a physician was called. Said he had worms; administered some cathartic and anthelmitic medicine, without any marked success.

The patient was put on the following treatment:

| | | |
|---|---|---|
| ℞ | Sub. mu. hyd., | grs. vi. |
| | Dover's powders, | xij. |

Mix. Divide into six powders; one to be taken three hours apart.

This treatment was persisted in, with blister to the spine; refrigerant drinks *ad libitum*.

This patient succumbed, and died in forty-eight hours from my first visit. Autopsy could not be obtained.

Query—If it was not acute spinal meningitis, what was the disease?

